# Systolic [Ca^2+^]_i_ regulates diastolic levels in rat ventricular myocytes

**DOI:** 10.1113/JP274366

**Published:** 2017-07-23

**Authors:** Rajiv Sankaranarayanan, Kornél Kistamás, David J. Greensmith, Luigi A. Venetucci, David A. Eisner

**Affiliations:** ^1^ Unit of Cardiac Physiology, Division of Cardiovascular Sciences, Manchester Academic Health Sciences Centre University of Manchester Manchester UK; ^2^ Biomedical Research Centre, School of Environment and Life Sciences, Peel Building University of Salford Salford UK

**Keywords:** calcium, diastolic, sarcoplasmic reticulum

## Abstract

**Key points:**

- For the heart to function as a pump, intracellular calcium concentration ([Ca^2+^]_i_) must increase during systole to activate contraction and then fall, during diastole, to allow the myofilaments to relax and the heart to refill with blood.
- The present study investigates the control of diastolic [Ca^2+^]_i_ in rat ventricular myocytes.
- We show that diastolic [Ca^2+^]_i_ is increased by manoeuvres that decrease sarcoplasmic reticulum function. This is accompanied by a decrease of systolic [Ca^2+^]_i_ such that the time‐averaged [Ca^2+^]_i_ remains constant.
- We report that diastolic [Ca^2+^]_i_ is controlled by the balance between Ca^2+^ entry and Ca^2+^ efflux during systole.
- The results of the present study identify a novel mechanism by which changes of the amplitude of the systolic Ca transient control diastolic [Ca^2+^]_i_.

**Abstract:**

The intracellular Ca concentration ([Ca^2+^]_i_) must be sufficently low in diastole so that the ventricle is relaxed and can refill with blood. Interference with this will impair relaxation. The factors responsible for regulation of diastolic [Ca^2+^]_i_, in particular the relative roles of the sarcoplasmic reticulum (SR) and surface membrane, are unclear. We investigated the effects on diastolic [Ca^2+^]_i_ that result from the changes of Ca cycling known to occur in heart failure. Experiments were performed using Fluo‐3 in voltage clamped rat ventricular myocytes. Increasing stimulation frequency increased diastolic [Ca^2+^]_i_. This increase of [Ca^2+^]_i_ was larger when SR function was impaired either by making the ryanodine receptor leaky (with caffeine or ryanodine) or by decreasing sarco/endoplasmic reticulum Ca‐ATPase activity with thapsigargin. The increase of diastolic [Ca^2+^]_i_ produced by interfering with the SR was accompanied by a decrease of the amplitude of the systolic Ca transient, such that there was no change of time‐averaged [Ca^2+^]_i_. Time‐averaged [Ca^2+^]_i_ was increased by β‐adrenergic stimulation with isoprenaline and increased in a saturating manner with increased stimulation frequency; average [Ca^2+^]_i_ was a linear function of Ca entry per unit time. Diastolic and time‐averaged [Ca^2+^]_i_ were decreased by decreasing the L‐type Ca current (with 50 μm cadmium chloride). We conclude that diastolic [Ca^2+^]_i_ is controlled by the balance between Ca entry and efflux during systole. Furthermore, manoeuvres that decrease the amplitude of the Ca transient (without decreasing Ca influx) will therefore increase diastolic [Ca^2+^]_i_. This identifies a novel mechanism by which changes of the amplitude of the systolic Ca transient control diastolic [Ca^2+^]_i_.

## Key points

- For the heart to function as a pump, intracellular calcium concentration ([Ca^2+^]_i_) must increase during systole to activate contraction and then fall, during diastole, to allow the myofilaments to relax and the heart to refill with blood.
- The present study investigates the control of diastolic [Ca^2+^]_i_ in rat ventricular myocytes.
- We show that diastolic [Ca^2+^]_i_ is increased by manoeuvres that decrease sarcoplasmic reticulum function. This is accompanied by a decrease of systolic [Ca^2+^]_i_ such that the time‐averaged [Ca^2+^]_i_ remains constant.
- We report that diastolic [Ca^2+^]_i_ is controlled by the balance between Ca^2+^ entry and Ca^2+^ efflux during systole.
- The results of the present study identify a novel mechanism by which changes of the amplitude of the systolic Ca transient control diastolic [Ca^2+^]_i_.

**Abbreviations**
[Ca^2+^]_i_: intracelleular Ca concentration
ISO: isoproterenol
NCX: sodium‐calcium exchange
RyR: ryanodine receptor
SERCA: sarco/endoplasmic reticulum Ca‐ATPase
SR: sarcoplasmic reticulum

## Introduction

For the heart to function as a pump, the intracellular Ca concentration ([Ca^2+^]_i_) must increase during systole to activate contraction and then fall, during diastole, to levels that are sufficiently low to allow the myofilaments to relax and the heart to refill with blood. Although the mechanisms that control the amplitude of the systolic rise of [Ca^2+^]_i_ are well understood (Bers, 2001), the regulation of the diastolic level is obscure (Louch *et al*. 2012). One problem is that much of the work studying [Ca^2+^]_i_ has used unphysiologically slow rates of stimulation. Under these conditions, a steady‐state is reached where Ca entry into the cell balances efflux and there is no flux into and out of the sarcoplasmic reticulum (SR) (Allen *et al*. 1984; Rios, 2010). By contrast, at faster rates, fluxes of Ca into and out of the SR will play an important role. Indeed, diastolic [Ca^2+^]_i_ increases with increasing rate (Layland & Kentish, 1999; Dibb *et al*. 2007).

An elevation of diastolic [Ca^2+^]_i_ has been reported in some studies of heart failure (Gwathmey *et al*. 1987; Beuckelmann *et al*. 1992; Sipido *et al*. 1998; Fischer *et al*. 2013). The frequency‐dependent increase of diastolic [Ca^2+^]_i_ (Gwathmey *et al*. 1991) and force (Pieske *et al*. 2002) is increased in heart failure in humans and this may contribute to the phenomenon of ‘diastolic heart failure’ (Selby *et al*. 2011). Raised diastolic [Ca^2+^]_i_ also increases Ca leak from the SR by increasing efflux of Ca through the ryanodine receptor (RyR) (Bovo *et al*. 2011) and raised levels as a result of RyR leak have been suggested to initiate a vicious cycle by increasing this leak further (Louch *et al*. 2012).

End diastolic [Ca^2+^]_i_ presumably depends on a combination of fluxes as a result of both sarcolemmal and SR Ca handling proteins. Heart failure impairs SR function as a result of a combination of a decrease of SERCA activity and increased RyR leak (Lou *et al*. 2012). Previous work has shown that decreasing SERCA activity elevates diastolic Ca (Negretti *et al*. 1993). In addition, making the RyR leaky with ryanodine elevates [Ca^2+^]_i_, particularly at high stimulation rates (Gao *et al*. 1995). However, the exact mechanism by which alterations of SR function affect diastolic [Ca^2+^]_i_ is unresolved. The aim of the present study was to investigate, quantitatively, the effects of interfering with the SR on diastolic [Ca^2+^]_i_ . We find that decreasing SR function decreases systolic and increases diastolic [Ca^2+^]_i_. Importantly, there is no effect on the average level of [Ca^2+^]_i_. We conclude that the increase of diastolic [Ca^2+^]_i_ is a consequence of the decrease of systolic [Ca^2+^]_i_ decreasing the efflux of Ca from the cell, and therefore that systolic [Ca^2+^]_i_ plays a major role in controlling diastolic [Ca^2+^]_i_.

## Methods

### Ethical approval

Animals were cared for and used in accordance with The UK Animals (Scientific Procedures) Act, 1986 and Directive 2010/63/EU of the European Parliament. The experiments were approved by the University of Manchester Ethical Review Board. Male Wistar rats (weighing ∼200–250 g) were killed by stunning and cervical dislocation. Single ventricular myocytes were isolated by digestion with collagenase and protease as described previously (Eisner *et al*. 1989).

Isolated myocytes were superfused with a solution (control) consisting of (in mm) 135 NaCl, 11.1 glucose, 1 CaCl_2_, 10 Hepes, 1 MgCl_2_ and 4 KCL. Then, 4‐aminopyridine (5 mm) and BaCl_2_ (0.1 mm) were added to inhibit K^+^ currents and the solution was titrated to pH 7.4 using NaOH. Probenecid (2 mm) was added to reduce loss of indicator from the myocytes. Micropipettes (< 5 MΩ) were filled with a solution consisting of (in mm): 125 KCH_3_O_3_S, 12 KCl, 10 NaCl, 10 Hepes, 5 MgCl_2_ and 0.1 EGTA; titrated to pH 7.2 with KOH; and a final concentration of amphotericin B of 240 g ml^−1^. Cells were voltage clamped with the perforated patch clamp technique using the discontinuous switch clamp mode (frequency 1–2 kHz and gain 0.3–0.7 nA mV^−1^) of an Axoclamp 2A voltage clamp amplifier (Molecular Devices, Union City, CA, USA). Cells were voltage clamped and stimulated at a range of frequencies (0.2 to 3 Hz) with a 40 mV, 100 ms duration pulse from a holding potential of −40 mV. All experiments were performed at room temperature.

### [Ca^2+^]_i_ measurements

Cells were incubated with the acetoxymethyl ester of Fluo‐3 (5 μm for 10 min) and allowed to de‐esterify before use. An aliquot was then placed in a superfusion chamber mounted on the stage of an inverted fluorescence microscope. To measure changes of [Ca^2+^]_i_, at the end of each experiment, the maximum fluorescence (*F*_max_) was measured by damaging the cell with the patch pipette. The dissociation constant of Fluo‐3 (*K*_d_) was taken as 864 nm (Cheng *et al*. 1993) and [Ca^2+^]_i_, calculated as described previously (Trafford *et al*. 1999).

Diastolic [Ca^2+^]_i_ was calculated by averaging [Ca^2+^]_i_ during the final 50 ms before the next stimulus. The amplitude of the Ca transient was calculated by subtracting diastolic [Ca^2+^]_i_ from peak [Ca^2+^]_i_. Average [Ca^2+^]_i_ was calculated as the mean level from one stimulus to the next. In some experiments, SR Ca content was estimated by releasing Ca from the SR using a mixture of 5 mm caffeine and 20 mm 2,3‐butanedione monoxime (Kashimura *et al*. 2010).

The Ca influx through the L‐type Ca current was calculated by integrating the Ca current (Venetucci *et al*. 2007). All analysis was performed using custom‐written software (Greensmith, 2014).

All chemicals were obtained from Sigma‐Aldrich (Poole, UK), R&D Systems (Abingdon, UK) or Fisher Scientific (Loughborough, UK). Caffeine was added as required. Ryanodine and thapsigargin were both stored as 1 mm stock solutions in DMSO and made up to a concentration of 1 μm before use. Thapsigargin was dissolved in DMSO and stored as a 1 mm stock solution and used at a final concentration of 1 μm.

### Statistical analysis

Data are reported as the mean ± SEM where applicable for descriptive analysis. Statistical comparisons were made using two‐way ANOVA. Regression lines were compared with an *F* test. *P* < 0.05 was considered statistically significant.

## Results

### Effects of RyR leak on diastolic and systolic [Ca^2+^]_i_

**Figure 1:**
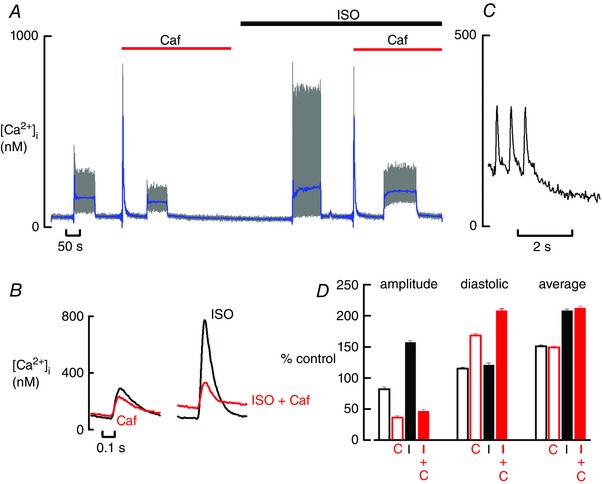
The effects of caffeine on systolic, diastolic and average [Ca^2+^]_i_ *A*, original timecourse. The grey trace shows [Ca^2+^]_i_ during rest and stimulation at 2 Hz. Caffeine (Caf, 1 mm) and ISO (1 μm) were applied as shown. The blue trace shows the average [Ca^2+^]_i_ for each transient. *B*, specimen, averaged (*n* = 20) transients. The left‐hand records were obtained in the absence and the right‐hand records are those in the presence of ISO. Red traces obtained in the presence of caffeine (1 mm). *C*, decay of [Ca^2+^]_i_ at the end of stimulation. The record shows the last three transients and the period afterwards from (*A*) in ISO + Caf. *D*, mean data from six experiments. Bars show data at a stimulation rate of 2 Hz. For each cell, the data have been normalized to the corresponding values obtained during stimulation at 0.5 Hz in the absence of both caffeine and ISO. The left‐hand group of four bars shows amplitude, the next group shows diastolic [Ca^2+^]_i_ and the right‐hand group shows the average [Ca^2+^]_i_. In each group, the unlabelled bar is the control; C, caffeine; I, ISO; I+C, ISO + caffeine. For clarity, statistical significance is not presented but is reported in the main text. [Color figure can be viewed at wileyonlinelibrary.com]

The first series of experiments examined the effect of increased RyR leak on the response to an increased stimulation rate. The grey traces in Fig. 1 *A* show the effect of periods of stimulation at 2 Hz. Under basal conditions, the diastolic level of [Ca^2+^]_i_ during the 2 Hz stimulation was only slightly greater than that during rest. The subsequent addition of caffeine (1 mm) to increase RyR leak decreased the amplitude of the Ca transient and modestly elevated diastolic [Ca^2+^]_i_. The effects of β‐adrenergic stimulation with isoprenaline (ISO) (1 μm) were then investigated on these phenomena. In agreement with previous work, ISO increased the amplitude of the systolic Ca transient (Hussain & Orchard, 1997); there was little effect on diastolic [Ca^2+^]_i_. However, when the cell was stimulated in the presence of caffeine plus ISO, the increase of diastolic [Ca^2+^]_i_ was greater than that in caffeine in the absence of ISO. These changes are seen in more detail in the expanded records of Fig. 1 *B*. The origin of the increase of diastolic Ca is revealed in Fig. 1 *C*. Increased leak slows the decay of the Ca transient such that, at elevated rates of stimulation, there is no time for decay to the resting level. In this example, the decay is biphasic, with a fast phase preceding the slow one. With more extreme leak, a slow monophasic decay is seen (Sankaranarayanan *et al*. 2016). The mean data (Fig. 1 *D*) show values normalized to those obtained at a slow stimulation rate (0.5 Hz) in the absence of both caffeine and ISO. It is clear that the effects of ISO on both systolic and diastolic [Ca^2+^]_i_ are greatly affected by the presence of caffeine. For example, in the absence of caffeine, ISO greatly increases the amplitude of the Ca transient (from 82 ± 3% to 157 ± 3% of control at 2 Hz stimulation, *P* < 0.001, ANOVA), whereas there is no effect on diastolic [Ca^2+^]_i_ (from 115 ± 2.5 nm to 121 ± 3.1%, *P* = 0.18). By contrast, in the presence of caffeine, ISO had a much smaller effect on the amplitude of the Ca transient (from 36 ± 3% to 46 ± 3%, *P* = 0.037) but markedly increased diastolic [Ca^2+^]_i_ (from 168 ± 3% to 208 ± 4%, *P* < 0.001).

### Effects of RyR leak on average [Ca^2+^]_i_

The above data show that caffeine increases diastolic [Ca^2+^]_i_ at the same time as decreasing the systolic rise of [Ca^2+^]_i_. Accordingly, we next investigated the effect of caffeine on time‐averaged [Ca^2+^]_i_ as shown by the blue trace in Fig. 1 *A*. It is clear that average [Ca^2+^]_i_ is: (i) increased by stimulation; (ii) increased by ISO; and (iii) unaffected by caffeine in both control and ISO. These observations are confirmed by the mean data of Fig. 1 *D*, which show that the average [Ca^2+^]_i_ does not significantly change upon addition of caffeine (control 151 ± 2% *vs*. caffeine 149 + 2%; *P* = 0.61; data normalized to 0.5 Hz stimulation). A similar finding is seen in the presence of ISO (ISO 208 ± 3% *vs* ISO + caffeine 212 ± 4%; *P* = 0.3). Finally, these data also show that ISO increases average [Ca^2+^]_i_ in both the presence and absence of caffeine (*P* < 0.001).

### The frequency‐dependence of average [Ca^2+^]_i_

**Figure 2:**
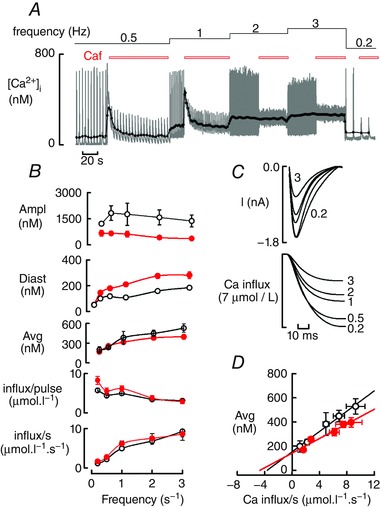
The frequency dependence of the effects of interfering with SR function on [Ca^2+^]_i_ *A*, timecourse of effects of caffeine. Stimulation frequency was altered and caffeine (Caf, 1 mm) applied as shown above. ISO (1 μm) was present throughout. The grey trace shows the original data and the dark trace is the average [Ca^2+^]_i_ on each transient. *B*, mean data showing frequency dependence. Mean data (14–20 cells) of the effects of frequency on the parameters (from top to bottom) are shown: amplitude of the Ca transient; diastolic [Ca^2+^]_i_; average [Ca^2+^]_i_; Ca influx per pulse; and Ca influx per second. Red symbols indicate the presence of caffeine. *C*, effects of stimulation frequency on the L‐type Ca current (absence of caffeine). Top, specimen Ca currents obtained at frequencies from 0.2 to 3 Hz. Bottom, timecourse of the integral of the Ca current to show Ca influx. For clarity, the full range of frequencies is only indicated on the integral traces. For the Ca currents, the extreme frequencies are indicated. For the other frequencies, the order of speed of inactivation of the L‐type Ca current parallels the simulation frequency. *D*, relationship between average [Ca^2+^]_i_ and Ca influx per second. Data taken from (*B*). Black symbols indicate the absence and red symbols indicate the the presence of caffeine. [Color figure can be viewed at wileyonlinelibrary.com]

Because the data of Fig. 1 show that the effects of caffeine on diastolic [Ca^2+^]_i_ were more prominent in the presence of ISO, all subsequent experiments were performed in the presence of ISO. The experiment shown in Fig. 2 was designed to investigate Ca handling over a wider range of frequencies. Figure 2 *A* shows the data obtained in the presence and absence of caffeine. As the frequency of stimulation increased, the Ca transient amplitude decreased slightly and diastolic [Ca^2+^]_i_ increased. The decrease in Ca transient amplitude was accompanied by (and is presumably at least in part caused by) a decrease in the amplitude of the L‐type Ca current (Fig. 2 *C*) (Antoons *et al*. 2002; Dibb *et al*. 2007). The effects of frequency were increased by caffeine. The continuous line in Fig. 2 *A* shows, again, that average [Ca^2+^]_i_ was unaffected by caffeine and was increased by increasing frequency. Notably, the increase of average [Ca^2+^]_i_ was a saturating function of frequency as shown by the fact that raising rate from 0.5 to 1 Hz had a larger effect than that from 2 to 3 Hz.

The above observations of the frequency dependence of average [Ca^2+^]_i_ are emphasized by the mean data shown in Fig. 2 *B*. Caffeine decreased the amplitude of the Ca transient at all frequencies. An increase of frequency increased diastolic [Ca^2+^]_i_; this effect is much more obvious in the presence of caffeine. By contrast to the marked effects of caffeine on both diastolic and systolic [Ca^2+^]_i_, average [Ca^2+^]_i_ was unaffected by caffeine (ANOVA, *P* > 0.5 at all frequencies). Average [Ca^2+^]_i_ did, however, increase in a saturating manner with an increasing frequency of stimulation. Figure 2 *B* sheds light on this saturation of average Ca. The Ca influx via the L‐type Ca current on each pulse decreased with an increasing stimulation rate (Fig. 2 *C*) as a result of increasing inactivation (Antoons *et al*. 2002; Dibb *et al*. 2007). Consequently, the Ca influx per unit time (Fig. 2 *B*, bottom) was a saturating function of frequency and therefore paralleled the frequency dependence of average [Ca^2+^]_i_. Data obtained in the presence of caffeine (Fig. 2 *B*, red symbols) were identical to those in its absence (ANOVA, *P* > 0.5 at all frequencies). The correlation between Ca entry per unit time and average [Ca^2+^]_i_ is emphasized by Fig. 2 *D*, which shows a clear linear relationship between these two parameters that is not statistically different (*F* test; *P* = 0.064) in the absence and presence of caffeine.

### The effects of thapsigargin and ryanodine

**Figure 3:**
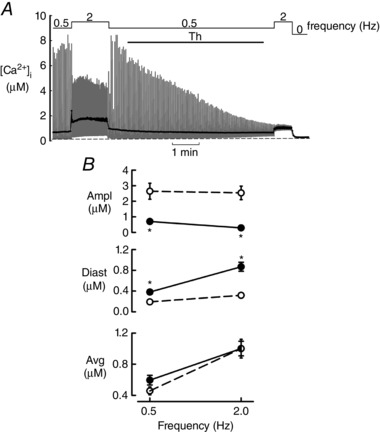
The effects of interfering with SR Ca handling with thapsigargin on [Ca^2+^]_i_ during stimulation All experiments were performed in the presence of ISO (1 μm). *A*, effects of thapsigargin. Thapsigargin (Th) (1 μm) was applied for the period shown above. The cell was stimulated at the frequencies indicated below. *B*, mean data from 10–12 experiments at stimulus rates of 0.5 and 2 Hz. From top to bottom: amplitude, diastolic and average [Ca^2+^]_i_. Open symbols before and closed in presence of thapsigargin. ^*^ *P* < 0.05 (two‐way repeated measures ANOVA) for comparisons between control and thapsigargin (ANOVA performed on the nine cells that had measurements at both frequencies).

**Figure 4:**
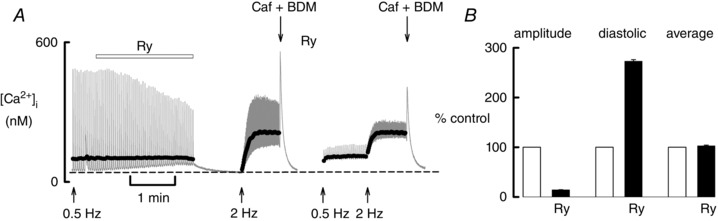
The effects of ryanodine *A*, timecourse of the effects of ryanodine (1 μm) on [Ca^2+^]_i_ studied at rest, 0.5 and 2 Hz. *B*, mean data from six experiments. Bars show data at a stimulation rate of 2 Hz. For each cell, the data have been normalized to the corresponding values obtained during stimulation in the absence of ryanodine. The left‐hand two bars show the amplitude, the next two show diastolic [Ca^2+^]_i_ and the right‐hand bars show the average [Ca^2+^]_i_. In each group, the unlabelled, open bar is the control and the filled bar is ryanodine (Ry).

The purpose of the experiments shown in Figs 3 and 4 was to examine whether the above observations were specific to caffeine or were a general consequence of interfering with RyR function. In the experiment shown in Fig. 3 *A*, the application of thapsigargin decreased systolic [Ca^2+^]_i_ and increased diastolic [Ca^2+^]_i_ during stimulation at 0.5 Hz. On increasing the stimulation rate to 2 Hz, there was a more marked increase of diastolic and decrease of systolic [Ca^2+^]_i_. When stimulation was stopped, [Ca^2+^]_i_ declined to a level similar to the original diastolic one. The mean data of Fig. 3 *B* show the effects of thapsigargin at 0.5 and 2 Hz. (Because thapsigargin is irreversible, it was not feasible to study the full range of frequencies used for caffeine). Thapsigargin decreased the amplitude and increased diastolic [Ca^2+^]_i_ at the same time as having no effect on average [Ca^2+^]_i_.

In the experiment shown in Fig. 4 *A*, the application of ryanodine decreased systolic [Ca^2+^]_i_ and increased diastolic [Ca^2+^]_i_. The dark line shows that average [Ca^2+^]_i_ remained constant during this period. When stimulation was stopped, [Ca^2+^]_i_ declined to a level similar to the original diastolic one. Subsequent stimulation at 2 Hz increased average [Ca^2+^]_i_. Ryanodine was reapplied to increase its effect. When stimulation was recommenced at 0.5 Hz, the Ca transient was considerably smaller than that observed previously at this frequency. The average [Ca^2+^]_i_ was, however, almost identical. Increasing the stimulation rate to 2 Hz resulted in a smaller Ca transient than that seen at 2 Hz before. Once again, the average Ca was unaffected. These data were interrupted by exposures to caffeine plus 2,3‐butanedione monoxime to release Ca from the SR and thereby obtain an estimate of the fall of SR Ca content. The mean data of Fig. 4 *B* confirm that ryanodine decreases the amplitude of the Ca transient (to 14.1 ± 0.7%, *P* < 0.001) and increases diastolic [Ca^2+^]_i_ (to 273 ± 3.1%, *P* < 0.001), whereas there is no effect on average [Ca^2+^]_i_ (102.8 ± 1.4%; *P* = 0.17).

### Effects of decreasing Ca influx

**Figure 5:**
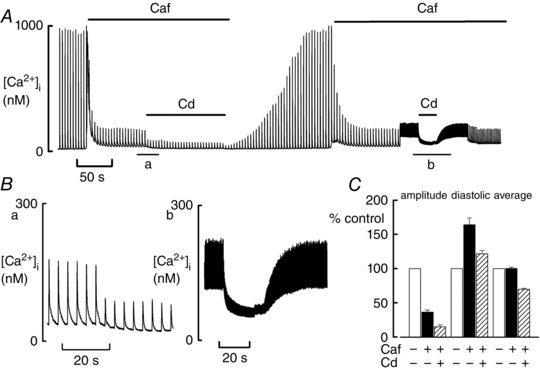
Effects of cadmium on diastolic [Ca^2+^]_i_ *A*, time course. The cell had been exposed to ISO (1 μm). Caffeine (Caf, 1 mm) and cadmium (Cd) (50 μm) were applied for the periods shown. The stimulation rate was 0.5 Hz until it was elevated to 3 Hz as shown. *B*, expanded records of the periods of application of Cd during stimulation at 0.5 (*Ba*) and 3 (*Bb*) Hz. *C*, Mean (*n* = 5 cells) data showing effects of Caf alone and Caf + Cd on amplitude, diastolic and average [Ca^2+^]_i_ at 0.5 Hz.

The above experiments suggest that the level of diastolic [Ca^2+^]_i_ depends on a balance between Ca influx and efflux. If this is the case, diastolic [Ca^2+^]_i_ would be expected to be decreased by reducing Ca influx. In the experiment shown in Fig. 5 *A*, caffeine had been added when stimulating at either 0.5 or 3 Hz. In agreement with the results reported above, the rise of diastolic [Ca^2+^]_i_ was greater at the higher frequency. Addition of cadmium chloride (50 μm) to decrease the L‐type Ca current was observed to decrease diastolic [Ca^2+^]_i_ at both stimulation rates (Fig. 5 *B*). On average, cadmium decreased the L‐type Ca current to 33 ± 5% (*n* = 5 cells). Figure 5 *C* shows the mean data obtained from five cells (at 0.5 Hz). One‐way ANOVA showed that cadmium decreased average and diastolic [Ca^2+^]_i_, as well as the amplitude of the Ca transient (all *P* < 0.001). Specifically, cadmium decreased the average [Ca^2+^]_i_ to 69.6 ± 1.7%.

## Discussion

The present study investigates the control of diastolic [Ca^2+^]_i_ in rat ventricular myocytes. In agreement with previous work, we find that increasing stimulation frequency increases diastolic [Ca^2+^]_i_ (Layland & Kentish, 1999; Dibb *et al*. 2007). We also add the important findings: (i) diastolic [Ca^2+^]_i_ is increased by manoeuvres that decrease SR function, such as increased RyR leak or decreased SERCA activity; (ii) this is accompanied by a decrease of systolic [Ca^2+^]_i_, such that the time‐averaged [Ca^2+^]_i_ remains constant; (iii) time‐averaged [Ca^2+^]_i_ is increased by β‐adrenergic stimulation and is a saturating function of frequency reflecting a frequency‐dependent decrease of Ca influx per beat; and (iv) diastolic [Ca^2+^]_i_ is regulated by the difference between the Ca influx and the systolic efflux on each beat.

### The effects of interfering with SR function on diastolic [Ca^2+^]_i_

Previous work has shown that ryanodine and thapsigargin increase diastolic [Ca^2+^]_i_ (Hansford & Lakatta, 1987; Negretti *et al*. 1993). In the present study, we clarified the mechanism of this effect and found that the reduction in Ca transient caused by increasing leak with caffeine or ryanodine was always associated with an increase in diastolic [Ca^2+^]_i_. The same behaviour was observed after the application of thapsigargin to inhibit SERCA. In other words, decreased systolic [Ca^2+^]_i_ associated with increased diastolic [Ca^2+^]_i_ is a distinguishing feature of impaired SR function, no matter whether this results from increased RyR leak or decreased SERCA activity. In the absence of stimulation, interfering with the SR had no effect on the level of resting [Ca^2+^]_i_ (Figs 1, 2 and 4). This is to be expected because, under these conditions, [Ca^2+^]_i_ is presumably determined solely by the surface membrane (Allen *et al*. 1984; Rios, 2010). The increase of diastolic [Ca^2+^]_i_ at elevated frequencies results from the fact that the next transient begins before the previous one has relaxed. Increasing leak or decreasing SERCA slows the rate of relaxation of the Ca transient (Negretti *et al*. 1993; Belevych *et al*. 2007; Sankaranarayanan *et al*. 2016). A combination of increased frequency and slowed relaxation would be expected to elevate diastolic [Ca^2+^]_i_. The frequency‐dependence of diastolic [Ca^2+^]_i_ is particularly obvious for thapsigargin (Fig. 3 *B*). This may be because inhibition of SERCA greatly slows the decay of the Ca transient. At low rates, the increased duration of the Ca transient compensates for the decreased amplitude in maintaining efflux such that diastolic [Ca^2+^]_i_ does not increase. This is impossible at high rates because the decay of the transient is interrupted by the next stimulus.

A key question is what causes the inverse relationship between diastolic and systolic [Ca^2+^]_i_ such that average [Ca^2+^]_i_ is maintained constant when SR function is altered? We suggest that the answer resides in the mechanisms that maintain Ca flux balance. In the steady‐state, during each cycle of stimulation, the influx mediated by the Ca current must be precisely equal to the Ca efflux, largely via sodium‐calcium exchange (NCX) (Eisner *et al*. 2013). The activity of NCX depends on [Ca^2+^]_i_. Increasing SR leak or decreasing SERCA activity will decrease the SR Ca content and therefore the amplitude of the systolic Ca transient. This, in turn, will decrease Ca efflux to a level less than the influx. Consequently, the cell will gain Ca and, because the SR is compromised, much of this Ca will remain in the cytoplasm, increasing diastolic [Ca^2+^]_i_. This elevated diastolic [Ca^2+^]_i_ produces more Ca efflux and compensates for the loss of efflux associated with the systolic transient (Dibb *et al*. 2007). If we assume that NCX activity is proportional to [Ca^2+^]_i_ then the Ca efflux per cycle will be proportional to average [Ca^2+^]_i_. If Ca influx is unaffected, then the need for constant efflux requires that average [Ca^2+^]_i_ be constant and therefore the decrease of systolic [Ca^2+^]_i_ must be balanced by an increase of diastolic such that average [Ca^2+^]_i_ remains constant.

This consideration of flux balance is a more complicated and general version of previous work showing that potentiating the opening of the RyR with low concentrations of caffeine leads to a transient increase of the amplitude of the systolic Ca transient (Trafford *et al*. 2000; Greensmith *et al*. 2014). In the steady‐state, however, the amplitude of the Ca transient was the same as in control. Under the conditions of those experiments (performed at low rates of stimulation), diastolic [Ca^2+^]_i_ did not change and therefore maintenance of flux balance required that systolic [Ca^2+^]_i_ was constant. In the present experiments, the changes of systolic [Ca^2+^]_i_ required that diastolic [Ca^2+^]_i_ change to maintain flux balance.

### The effects of β‐adrenergic stimulation on [Ca^2+^]_i_

The above analysis also explains why ISO increases average [Ca^2+^]_i_. ISO will increase Ca entry through the L‐type current and this will have to be balanced by increased efflux on NCX. This increased efflux can be achieved by an increase of average [Ca^2+^]_i_. The exact circumstances will determine whether the increase of average [Ca^2+^]_i_ results primarily from a rise of diastolic as opposed to systolic [Ca^2+^]_i_. For example (Fig. 1), with normal SR function, the increase of the amplitude of the systolic Ca transient is sufficiently large that diastolic [Ca^2+^]_i_ does not increase. By contrast, when the SR is partly disabled, systolic [Ca^2+^]_i_ cannot increase sufficiently and a rise of diastolic [Ca^2+^]_i_ ensues (Fig. 1 *D*). It is also possible that an increase of Ca leak from the SR, possibly via a Ca/calmodulin‐dependent kinase II (CaMKII) mechanism (Curran *et al*. 2007), contributes to the increase of diastolic [Ca^2+^]_i_. In this context, it is worth noting that, in mice overexpressing CaMKII, the rise of systolic [Ca^2+^]_i_ produced by ISO was less than in wild‐type. However, ISO produced a larger increase of diastolic [Ca^2+^]_i_ in the overexpressing mice compared to the control (Sag *et al*. 2009). Given that these transgenic mice have elevated SR Ca leak, the reciprocal effect on diastolic and systolic [Ca^2+^]_i_ is consistent with the conclusions of the present study. It should also be noted that the normal inotropic response to ISO, resulting from an increase of systolic [Ca^2+^]_i_ with no change of diastolic [Ca^2+^]_i_, requires a normal, functional SR. Because SR activity is compromised, an increase of diastolic [Ca^2+^]_i_ will occur.

### The effects of stimulation frequency

An increase of stimulation frequency will increase Ca influx per unit time, thereby requiring an increase of average [Ca^2+^]_i_ to maintain flux balance. From first principles, this can be achieved by an increase of either or both diastolic or systolic [Ca^2+^]_i_. In the present experiments, performed on rat myocytes, increased frequency decreases systolic [Ca^2+^]_i_ and therefore systolic efflux. As the frequency is increased, the cell therefore faces three challenges: (i) increased Ca influx per unit time (Fig. 2 *B*); (ii) decreased systolic efflux; and (iii) decreased diastolic time for efflux to occur in. Ca flux balance can therefore only be established with an increase of diastolic [Ca^2+^]_i_. If SR function is depressed, increased frequency produces a larger decrease of systolic [Ca^2+^]_i_ and therefore a larger rise of diastolic [Ca^2+^]_i_ would be expected. In agreement with these predictions, we find that increasing frequency increases diastolic [Ca^2+^]_i_ and this increase is potentiated by increasing SR leak or decreasing SERCA activity. Average [Ca^2+^]_i_ is a saturating function of frequency (Fig. 2 *B*) presumably because the Ca entry per unit time also saturates with frequency as a result of increased inactivation of the L‐type Ca current (Fig. 2 *C*), (Antoons *et al*. 2002; Dibb *et al*. 2007). Consistent with this, average [Ca^2+^]_i_ is a linear function of Ca influx per unit time through the L‐type Ca current (Fig. 2 *D*). It should be noted that, if the L‐type Ca current did not decrease at higher frequencies, the rise of diastolic [Ca^2+^]_i_ would be even greater.

One final conclusion can be derived from Fig. 2 *D*. It is clear that, even with zero influx through the L‐type Ca current, [Ca^2+^]_i_ has a finite value. This has been accounted for by a background Ca entry (Choi *et al*. 2000; Kupittayanant *et al*. 2006) which is unaffected by stimulation rate. The value of this background flux, estimated from the horizontal intercept of Fig. 2 *D* is of the order of 4 μmol l^−1^ s^−1^. The existence of this background flux may also be relevant to the effects of cadmium. We found that 50 μm cadmium decreased Ca influx to 33% but average [Ca^2+^]_i_ fell to only 70%. This discrepancy can be accounted for if the background flux is unaffected by cadmium.

### Limitations

It should be noted that, in these experiments, we used a holding potential of −40 mV to inactivate the Na^+^ current. This holding potential will decrease the L‐type Ca current and lead to an underestimate of the effects of frequency on diastolic [Ca^2+^]_i_ (Dibb *et al*. 2007). The removal of Na^+^ current might be expected to decrease the frequency‐dependent increase of intracellular Na^+^ concentration but, because Na^+^ influx through Na channels is quantitatively smaller than that through NCX (Bers *et al*. 2003), this may not be a major issue. Previous work has measured systolic and diastolic [Ca^2+^]_i_ in rat ventricular myocytes excited with physiological action potentials. An increase of frequency increased both diastolic and systolic [Ca^2+^]_i_ indicated that a frequency‐dependent increase of average [Ca^2+^]_i_ is also seen with more physiological stimulation (Dibb *et al*. 2007). As a more general point, the above discussion assumes that the only factor regulating NCX is [Ca^2+^]_i_. It therefore ignores the effects that changes of intracellular sodium concentration ([Na^+^]_i_) may have on NCX activity. [Na^+^]_i_ will be increased by an increase of stimulation rate and as a consequence of the increased NCX activity in response to the Ca loading produced by β‐adrenergic stimulation. It will be decreased as a consequence of phosphorylation of phospholemman and stimulation of the sodium pump (Bers *et al*. 2003). It should also be noted that the above discussion is based on the assumption that NCX activity is proportional to [Ca^2+^]_i_ . This will only be true over a certain range and, at higher [Ca^2+^]_i_, efflux will tend towards saturation. In this case, when SR function is decreased, a reduction of average [Ca^2+^]_i_ might be expected. Any such effect is below the resolution of the experiments. Finally, although, in the present experiments, Ca influx through the L‐type current was unaffected by altering SR function with caffeine, more generally, it is possible that changes of Ca‐dependent inactivation may affect the L‐type Ca current and this would need to be allowed for,

### Relationship with disease

Some previous work has shown that heart failure results in an increase of diastolic force and/or [Ca^2+^]_i_ at elevated rates of stimulation (Sipido *et al*. 1998; Baartscheer *et al*. 2003; Selby *et al*. 2011). One explanation for this is the measured increase of [Na^+^]_i_ which will decrease Ca efflux on NCX (Pieske *et al*. 2002). The results of the present study suggest an additional explanation for the rise of diastolic [Ca^2+^]_i_. It is known that heart failure is often associated with increased RyR leak (Marks, 2000; Marx *et al*. 2000; Shannon *et al*. 2003; Terentyev *et al*. 2008; Belevych *et al*. 2013) and decreased SERCA activity (Nagai *et al*. 1989; Mercadier *et al*. 1990; Hasenfuss *et al*. 1994). As a consequence of the resulting decrease of SR Ca content and hence systolic [Ca^2+^]_i_, these changes would be expected to also elevate diastolic [Ca^2+^]_i_. Indeed, both this mechanism and the increase of [Na^+^]_i_ will decrease Ca efflux and increase diastolic [Ca^2+^]_i_.

A common observation is that, in human heart failure, the reduction of the amplitude of the Ca transient is more marked at higher frequencies (Gwathmey *et al*. 1990; Mulieri *et al*. 1992). This is accompanied by removal of the increase of SR Ca content produced by increasing stimulation frequency (Lindner *et al*. 1998; Pieske *et al*. 1999). These effects have previously been attributed to a decrease of SERCA activity (Pieske *et al*. 1995). Our data suggest that Ca leak may also contribute to the loss of this frequency dependence in heart failure. The results of the present study are also relevant to the changes produced by increasing Ca buffering by the myofilaments as occurs in some case of hypertrophic cardiomyopathy. Such increased buffering slows the rate constant of decay of the Ca transient and increases diastolic [Ca^2+^]_i_ (Schober *et al*. 2012). The increased buffering will decrease the increase of [Ca^2+^]_i_ produced by a given total Ca release from the SR, thereby decreasing Ca efflux. This, and the slowed decay will elevate diastolic [Ca^2+^]_i_.

### Conclusion: systolic [Ca^2+^]_i_ controls diastolic [Ca^2+^]_i_

The results of the present study show that the time‐averaged level of [Ca^2+^]_i_ is an important factor in regulating Ca cycling. This average level determines the Ca efflux from the cell required to balance Ca influx. The total efflux can be considered to comprise two components: (i) a component activated by the diastolic level of [Ca^2+^]_i_ and (ii) an additional component that occurs during the systolic Ca transient (Dibb *et al*. 2007). Anything that decreases the amplitude of the systolic Ca transient, without affecting Ca influx, will decrease the systolic efflux, thereby requiring an increase of diastolic [Ca^2+^]_i_ to maintain Ca flux balance. In this way, the systolic Ca transient plays a vital role in regulating diastolic [Ca^2+^]_i_.

## Additional information

### Competing interests

The authors declare that they have no competing interests.

### Author contributions

DAE and LV designed the work. RS and KK performed the experiments. RS, KK and DJG analysed and interpreted the data. DAE, RS and LV wrote the manuscript. All authors revised the manuscript critically for important intellectual content. All authors approved the final version of the manuscript. All the experiments were carried out at The University of Manchester.

### Funding

This work was supported by grants from the British Heart Foundation to DAE and LAV (grant numbers: FS/10/063/28374; FS/11/15/28693; CH/2000004/12801).
